# Immunonutrition: facilitating mucosal immune response in teleost intestine with amino acids through oxidant-antioxidant balance

**DOI:** 10.3389/fimmu.2023.1241615

**Published:** 2023-09-29

**Authors:** Karina L. Hissen, Wenliang He, Guoyao Wu, Michael F. Criscitiello

**Affiliations:** ^1^ Comparative Immunogenetics Laboratory Department of Veterinary Pathobiology, Texas A&M University, College Station, TX, United States; ^2^ Amino Acid Laboratory, Department of Animal Science, Texas A&M University, College Station, TX, United States; ^3^ Department of Microbial Pathogenesis and Immunology, College of Medicine, Texas A&M Health Science Center, Texas A&M University, Bryan, TX, United States

**Keywords:** teleost, immunology, amino acid, antioxidant, reactive oxygen species

## Abstract

Comparative animal models generate fundamental scientific knowledge of immune responses. However, these studies typically are conducted in mammals because of their biochemical and physiological similarity to humans. Presently, there has been an interest in using teleost fish models to study intestinal immunology, particularly intestinal mucosa immune response. Instead of targeting the pathogen itself, a preferred approach for managing fish health is through nutrient supplementation, as it is noninvasive and less labor intensive than vaccine administrations while still modulating immune properties. Amino acids (AAs) regulate metabolic processes, oxidant-antioxidant balance, and physiological requirements to improve immune response. Thus, nutritionists can develop sustainable aquafeeds through AA supplementation to promote specific immune responses, including the intestinal mucosa immune system. We propose the use of dietary supplementation with functional AAs to improve immune response by discussing teleost fish immunology within the intestine and explore how oxidative burst is used as an immune defense mechanism. We evaluate immune components and immune responses in the intestine that use oxidant-antioxidant balance through potential selection of AAs and their metabolites to improve mucosal immune capacity and gut integrity. AAs are effective modulators of teleost gut immunity through oxidant-antioxidant balance. To incorporate nutrition as an immunoregulatory means in teleost, we must obtain more tools including genomic, proteomic, nutrition, immunology, and macrobiotic and metabonomic analyses, so that future studies can provide a more holistic understanding of the mucosal immune system in fish.

## 1 Introduction

Animals have benefited comparative immunology for over a millennium by providing a scientific platform to better understand biological processes, with human and veterinary medicine only significantly diverging in the early 20th century (1, 2). Comparative animal models generate fundamental scientific knowledge on immune responses, and mammals have been favored for those studies for their similarities with human biochemical mechanisms and physiology. To further expand our understanding of immunity, research in lower vertebrates such as zebrafish (*Danio rerio*) has rapidly gained popularity in immunology since 1997 (3), and has led to the use of other teleost species as models to investigate immune responses, particularly within the intestine. One component of interest is mucus, which is composed of antimicrobial peptides (AMPs), bactericidal enzymes, immunoglobulins, mucins, glycoproteins, and immune cells (4). Although fish may experience different pathogens compared with humans and other mammals, they are valuable models for intestinal inflammatory responses with many immune genes and mechanisms conserved from fish to mammals (3, 5, 6). Teleost fish dominate the species raised on aquaculture farms and when using these species as immunology models, we can further improve fish health via both animal science and veterinary biomedical research. Ultimately, research in multiple teleost species is crucial to fully investigate the natural history of biochemical mechanisms that can be applied to human biomedical research as well.

Farmed fish is a popular protein source for human consumption, and the Food and Agriculture Organization (FAO) reported that production in 2020 reached 87.5 million tonnes. This is higher than previous years and reduces the need for capture fisheries (7). The COVID-19 pandemic drove aquaculture production in the last few years due to restriction in fishing activities, with FAO projecting that production will reach 106 million tonnes by 2030 (7). To meet market demands and environment changes, sustainable farming techniques are used to improve the growth performance of fish within the industry (8, 9). Sustainable techniques for aquaculture are evaluated according to societal standards of impact on the environment, animal welfare, as well as safety for both the animals and workers (8, 10). Water quality and habitat are a sector of sustainable management to limit the spread of disease, especially in highly stocked farms (8, 9, 11).

Mucosal surfaces are in direct contact with the water environment to provide the first line of defense against pathogens. Currently, vaccines provide the best method to prevent infectious disease, as they can be efficient and cost-effective (12). However, the protection is limited to specific known pathogens and research is still identifying the most effective administration routes for existing vaccines in fish (13–15). Mucosal vaccines are available and have been explored for they are non-invasive and decrease the risks of exposure to pathogens; however, there are challenges with vaccine efficacy from administration (16–18). For long-term B and T memory responses to be strong at mucosal surfaces, the immunization needs to adhere to the surface and penetrate the mucus; however, mucosal surfaces dilute the immunization and, despite increasing the dosage, vaccines can be broken down by proteases present on these surfaces (18, 19). In the gut, one challenge is that the antigen often needs to break intestinal tolerance through stimulation of lymphocytes within the lamina propria of mucosal tissues (17, 20). The use of antibiotics in the aquaculture industry is another approach to control infections but can be expensive (21) and if misused, can lead to selection of bacteria with antibiotic resistance in humans (15).

Instead of targeting the pathogen itself, nutrition has become a preferred approach for managing fish health for it is less invasive or labor intensive than administering vaccines while still providing immune modulation properties to fight infections and limit losses from diseases (13, 22). Using nutrition to modulate fish immune responses can enhance immunological functions by targeting biochemical mechanisms for immunostimulation (13). Nutrient immunostimulants include vitamins, minerals, fatty acids, and amino acids (AAs), which all provide a non-toxic, non-invasive, and environmentally friendly method to support immune responses (23). These feed additives as immunostimulants can enhance the expression of genes related to humoral and cellular immunities (23, 24). Additionally, nutrient immunostimulants can regulate and shape immune responses by activating cytokine secretion, receptor expression and other components within the innate and adaptive immune systems (23, 25). AAs as nutrient immunostimulants are a niche of immunonutrition that depends on cellular requirements for an immune response. Cells require more metabolic fuels and nutrients during an infection, particularly AAs for protein synthesis until the pathogen is cleared (13). The availability of nutrients can impact oxidative-antioxidant balance by promoting the production of metabolites from AAs such as glutathione (GSH) and nitric oxide (NO). Amino acids are the building blocks of proteins including antioxidant enzyme systems such as superoxide dismutase (SOD) and are essential for physiological development of teleost fish (26). All AAs are required to produce immune cells and antibodies, implying that their deficiency can weaken the immune system. Supplemental AAs in diets can act as an immunostimulant to modulate immune responses partly via antioxidant mechanisms (27). In immunonutrition, morphological and transcriptional analyses have been excellent tools to understand the health status of the gut, which can be examined after feed supplementation to determine optimal immune capacity to prevent infections (Abdul 28). By understanding AA impact on gut immune health, diets can be modified to favor specific immune responses that will not lead to oxidative stress but instead promote homeostasis once the pathogen has been cleared.

In this review, we will evaluate select AAs and their metabolites in oxidant-antioxidant balance and their use to improve mucosal immune capacity as well as gut integrity. We will have a brief overview of teleost fish immunology, including both innate and adaptive responses within the intestine. We will then expand on a particular immune defense mechanism called oxidative burst, which produces reactive oxygen species (ROS) to destroy bacterial pathogens, and discuss how this mechanism is activated through stress that fish encounter within aquaculture. This will lead to a discussion of the relationship between oxidative stress and gut mucosal immune responses under various environmental stressors, and current studies utilizing specific AAs to modulate mucosal immune responses in teleost intestine. Lastly, we will provide our perspectives on overcoming the experimental challenges in AA immunonutrition for more effective modulation of teleost gut immunity through oxidant-antioxidant balance.

## 2 Teleost fish immunology

### 2.1 Overview of mucosal and intestinal immune response

Although fish lack organized lymphoid structures in their gut, the gut wall contains a range of immune cells such as plasma cells, other lymphocytes, and antigen-presenting cells (APCs) (17, 29). The mucosal barrier maintains homeostasis in the organism through local immune responses to sustain tolerance with mutual and commensal bacteria (30). Differentiated epithelial cells called goblet cells within the mucosa prevent pathogen entry by secreting mucins, high molecular weight glycoproteins that can contain antimicrobial properties. Goblet cells are a typical morphological marker to identify active mucosal immune responses, for example in brown trout (*Salmo trutta*) during parasitic infections, the secreted mucus contains acidic glycoconjugates that increase mucus viscosity for intestinal protection (31). Intraepithelial lymphocytes (IELs) and lamina propria lymphocytes (LPLs) are lymphocytes that reside in the epithelial and lamina propria layers respectively, and recognize antigens to induce an adaptive immune response to maintain gut homeostasis and kill the pathogen (32).

The gastrointestinal tract of teleost fish has evolved to their aquatic lifestyle and feeding habits, but it is important to note that not all teleost fish have a stomach. For instance, zebrafish and common carp (*Cyprinus carpio*) lack stomachs and instead have an anterior intestine called the intestinal bulb, whereas fish like Nile tilapia (*Oreochromis niloticus*) and Atlantic cod (*Gadus morhua*) have a true stomach (3). The intestine is divided into three main regions: the pyloric caeca, midgut, and hindgut, which all aid in food digestion, nutrient absorption, and mucosal immunity. From their investigation in rainbow trout (*Onchorhynchus mykiss*) intestine, Verdile et al. (32) reported that gut segments should be accounted for when determining the relationship between metabolism and immune response. Typically, histological parameters used to determine gut health in response to dietary supplementation are based on classical morphological changes in the fish fed experimental and control diets, including villus length, lamina propria height, quantity of IELs, and qualitative changes in mucus secretion (32, 33). Differences in segment morphology could result from commensal bacteria populations within specific segments affecting the morphology of the brush border. Ultimately, these morphological changes can impact the expression of genes encoding immune signaling factors like chemokines and cytokines, which could lead to alterations in cellular signaling for immune defense. This is because enterocytes undergo rapid turnover to function properly and an imbalance between cell proliferation and apoptosis can lead to tissue dysfunction plus damage to the intestinal morphology (34).

Besides morphological analyses of the gut, other biomarkers present valuable information to better understand mechanisms impacted from supplementation. For example, intestinal alkaline phosphatase (ALP) activity is a biomarker for mature enterocytes and can be used in conjunction with the immunohistochemical localization of proliferating cell nuclear antigen (PCNA), where PCNA signal confirms the presence of stem cells (33). These signals tend to be within intestinal folds of the villi, and when identified with ALP activity, mature differentiated epithelial cells can be recognized (33). Alternatively, a lack of ALP leads to increased sensitivity to microbes and high levels of neutrophils, which modulate the composition and function of the gut microbiota (3). If pathogens have the ability to affect morphology as well as microbiota populations throughout the length of the gut, experiments should be cognizant of their impact on immune-relevant gene expression that alter mucosal defense (35). Published articles often either sample one gut segment or multiple segments, so validation is needed for past work utilizing only a single segment or less-defined ones to fully understand mucosal immunity throughout the gut length.

Production of adenosine triphosphate (ATP) and nicotinamide adenine dinucleotide phosphate (NADPH) depends on oxygen to fuel the mitochondria to meet the cell’s energy needs in an immune response (36). Circulating oxygen diffuses into tissues through the oxygen cascade gradient and functions as a regulator of ROS generation in the mitochondria (36). The mucosal barrier maintains homeostasis through the selective transport of nutrients including electrolytes and water from the intestinal lumen into the bloodstream while disposing of harmful substances to prevent them from entering the internal environment (34, 37, 38). Tight junctions, for example, can regulate the mucosal barrier by directly preventing toxins from entering the lumen. Development of the disease is closely associated with tight junction structure damage of intestinal epithelial cells that are triggered by pro-inflammatory cytokine released by the activation of the intestinal mucosal immune system (39–41). Tumor necrosis factor α (TNF-α) is a pro-inflammatory cytokine that can disrupt the stability of tight junction, thereby introducing oxidative stress (41). Wu et al. (42), concluded from their work on Songpu mirror carp (C*yprinus carpio Songpu)* that during *Aeromonas hydrophila* exposure, mRNA expression of tight junction structural protein genes was upregulated in intestinal mucosal cells to reduce intestinal permeability (42). This suggests that the regulation of mucosal tight junctions is to protect the integrity of the gut by controlling its environment and reduce oxidative damage. Intestinal epithelial cells communicate with the mucosa to facilitate an effective immune response, and this process starts with cellular signaling within the innate immune system.

### 2.2 Innate immune response

Innate immune cells and related proteins play crucial roles to neutralize pathogens. The process begins with the recognition of pathogen-associated molecular patterns in fish gut (PAMPs) by pattern recognition receptors (PRRs) expressed on gut epithelial cells (13). This interaction detects and recognizes pathogens that disrupt homeostasis and initiates chemical, biological, and/or mechanical responses to prevent further infection (43). Toll-like receptors (TLRs) are a type of PRR that have been evolutionarily conserved to mediate innate immune defense, recognizing PAMPs such as lipopolysaccharides (LPS), a component of bacterial cell walls. In fish, LPS activates the expression of inflammatory cytokine genes, such as the interleukins, IL-1β, IL-8, IL-17A/F, and TNF-α, likely mediated by TLR4 (44). TLR-4 is an important receptor for inflammation because it activates the nuclear factor kappa B (NF-κB) pathway, leading to the release of inflammatory mediators, including chemokines and cytokines (42, 45, 46). Conversely, some fish do not present TLR-4 or an ortholog to recognize LPS (47, 48). A dual-luciferase reporter assay was used to compare NF-κB activation in gilthead seabream (*Sparus aurata*), spotted green pufferfish (*Tetraodon nigroviridis*), and zebrafish showed that LPS signaled a TLR4 independently from the MyD88 pathway (47). The MyD88 is a signaling protein for TLRs induced by NF-κB for immune signaling and interestingly the zebrafish TLR4 orthologs negatively regulated the MyD88-dependent pathway, suggesting that TLR4 is a negative regulator to provide resistance to bacterial endotoxins (47, 48). Other TLRs have been identified in teleost species such as rock cod (*Eleginops maclovinus*) (49). Different portions of the intestine were investigated following exposure to the bacterium *Francisella noatunensis* and modulated the transcription of innate PRRs TLR1, TLR5 and TLR8 were observed. The transcriptional profiles of the evaluated TLRs were likely based on the binding capabilities of TLR1 to bacterial lipopeptides and LPS, TLR5 to flagellin, and TLR8 to single-stranded bacterial RNA (49, 50). These findings showed that TLR1, TLR5, and TLR8 recognized *F. noatunensis* in different parts of the gut at different levels, further supporting that the receptors on the gut following infection modulate transcription differently amongst the length of the intestine (49). Activation of specific TLRs can cause downstream immune effects based on the cytokines and chemokines being produced.

Notable cytokines that can attract phagocytic cells to the site of infection include IL-1β, a proinflammatory cytokine produced by monocytes/macrophages that signals the proliferation of leukocytes and downstream production of additional chemokines and other cytokines (51). IL-2 is an example of a downstream cytokine that can further promote inflammation through increased signaling of IL- 1β and NO production to recruit neutrophils, while stimulating T cells of adaptive immunity (51, 52). IL-8 is a pro-inflammatory chemokine that induces neutrophil and macrophage migration during oxidative stress or when infected by a pathogen to clean cellular debris and generate new tissue (51, 53). IL-17A/F are cytotoxic cytokines that CD4^+^ helper T cells use to communicate inflammation through AMPs. IL-17A is produced by adaptive Th17 cells, inducing the expression of cytokines such as IL-1β, TNF-α, and IL-6 in yellow catfish (*Pelteobagrus fulvidraco*) (54). IL-17F is responsible for protecting epithelial cells against bacterial invasion, upregulating other proinflammatory cytokines such as TNF-α, a multifunctional proinflammatory cytokine that drives the proliferation of immunocytes and phagocytic activity. Hoseinifar et al. (51) reported that TNF-α could enhance the phagocytic ability, ROS activity, and apoptosis of granulocytes/monocytes, suggesting that the leukocyte phagocytosis mechanism could be regulated by TNF-α in rainbow trout (*Oncorhynchus mykiss)*. Research in isolated leukocytes of largemouth bass (*Micropterus salmoides*) confirmed TNF-α’s role in phagocytosis, leading to an enhancement in phagocytic ability, ROS activity, and apoptosis of granulocytes (55).

Monocytes, macrophages, and neutrophils are granulocytes that mediate non-specific immunity by promoting inflammation and phagocytosis. Within macrophages, there are two major subpopulations: M1 and M2 macrophages. M1 has pro-inflammatory properties and expresses TNFα, whereas M2 has anti-inflammatory properties from increased expression of TGF-β, an anti-inflammatory cytokine (30, 56, 57). Monocytes and macrophages predominantly produce ROS and NO intracellularly, while neutrophils produce these molecules both intracellularly and extracellularly (4, 58–60). NO is a cytotoxic molecule of macrophages that mediates inflammation (26), where inflammation can be categorized as acute or chronic. Acute inflammation involves a series of events resulting in increases in blood flow as well as monocyte and neutrophil trafficking (61, 62). Acute inflammation can be shut down by inhibiting proinflammatory pathways and activating anti-inflammatory cytokines, like TGF-β. If the pathogen persists, the inflammation can become chronic by increasing blood flow through an increase in capillary permeability resulting from high amounts of antinutritional factors due to inadequate feed formulation (62–64). Chronic inflammation can lead to cellular resource depletion, tissue damage, and fibrosis unless it is optimally regulated by anti-inflammatory responses to neutralize ROS (63, 65). Acute inflammation can be prevented by inhibiting proinflammatory pathways and activating anti-inflammatory cytokines, such as TGF-β. If the pathogen persists, the inflammation can become chronic by increasing blood flow through an increase in capillary permeability resulting from either high amounts of antinutritional factors or inadequate feed intakes of nutrients (62, 63, 66). Chronic inflammation can lead to cellular resource depletion, tissue damage, and fibrosis unless it is optimally regulated by anti-inflammatory responses to neutralize ROS (63, 65). The cellular signaling in response to changes in an infection from acute to chronic depends on the cytokine and chemokines secreted from these immune cells. Inflammasomes provide surveillance, monitoring the infection site to promote the recruitment of immune cells causing inflammation, followed by expression of genes for anti-inflammatory signals to restore homeostasis and destroy damaged cells through an intracellular death process defined as apoptosis (67, 68).

In both fish and mammals, cytokines and chemokines are secreted through the bloodstream interacting with hepatocytes (68). This initiates crosstalk with various plasma proteins to signal for immune pathways like the complement system to be upregulated (69–71, 71, 72). The complement system in mammals includes three pathways with series of cascades of the cleaving until a terminal complex is lysed (69, 71, 73). Most subunits of the complement system have been identified in teleost fish and share structural similarities at the protein level with mammalian orthologs (69). Interestingly, teleost fish have variable isoforms of some components, notably C3 (74); C4 (75), C7 (76), C8 (77), C2/CFB (78), CFH (79), and CR3 (77). The various isoforms suggest different specificities for cleavage activation; however, the complement components are understudied, including the mechanisms involved to activate systems such as kallikrein (69, 80). Within the blood coagulation pathway in mammals, there is an important cascade with serine proteases called the kinin-kallikrein cascade that participates in blood clotting, blood pressure regulation and inflammation (81). Wong and Takei (82) found that the teleost fishes Nile tilapia and rainbow trout shared very little similarity in homologous structures of kininogens with mammals and had some components missing at the genomic level (82). A particular enzyme responsible for the creation of large structures with a high binding affinity had been lost through evolution within the teleost group (82). Instead, teleost fish have analogous structures within the kallikrein cascade and have a unique lectin activity known as the kalliklectin cascade (83). Andreeva et al. (81), investigated Atlantic cod serum for intracellular proteins to understand this cascade further, and observed no mammal-like osmotically active albumin but found osmotically active proteins that functionally replace the albumin independently from osmoregulation (81). The results from this experiment suggest that teleost albumin function was lost in fish during evolution, but other intracellular proteins are needed to maintain osmotic homeostasis (83). Their results demonstrated that these other plasma proteins are involved with transport in binding lectin to pathogens through the complement system and in immune regulation (83). To further explore the roles of neutralization of pathogens, we will discuss a crucial defense mechanism of teleost fish against invading pathogens: oxidative burst.

#### 2.2.1 Oxidative (respiratory) burst and ROS

In teleost fish, ROS and NO (a reactive nitrogen species) defend against pathogens by causing oxidative damage to their DNA, proteins, and lipids (26). In erythrocytes and immunocytes of these animals, the mitochondria function as an oxygen regulator by generating ROS and using energy to synthesize antioxidants. In fish, molecules of ROS are formed via many biochemical reactions (including NADPH oxidase and SOD), whereas NO is produced from arginine (84). At metabolic rest, ROS yield is typically low until a stressor induces a fight or flight response via activating the protein kinase C signaling pathway (36, 85). The primary electron donor for superoxide generation is NADPH, which is formed mainly via the hexose monophosphate shunt in endotoxin or phorbol myristate acetate (PMA)-stimulated macrophages to perform oxidative burst (86). Oxidative burst is primarily seen in phagocytic leukocytes where its receptors interact with the pathogen (59). When the pathogen is engulfed, a phagosome undergoes a series of steps to fuse with a lysosome, in which the lysosome activates the NADPH oxidase complex on the extracellular membrane of the phagolysosome to produce 90% of cell-derived ROS in the mitochondria (36, 59, 87). Organisms lacking this complex can suffer from chronic infections, leading to hyper-inflammation that can be fatal (86, 88). The NADPH oxidase complex consists of several subunits, including p22phox, gp91phox, p47phox, p67phox, and Rac (89, 90). The subunit gp91phox is first stimulated during the complex’s activation, and electrons are transferred from NADPH to O_2_ within the cytosol of the cell, resulting in the production of ROS, such as superoxide anion (O_2_^-^) with the enzyme SOD (89, 90). Superoxide anion is converted to hydrogen peroxide with catalase (CAT) and can be further converted to water with peroxidase (89, 90). ROS must be removed by either enzymatic antioxidants including SOD, CAT, glutathione peroxidase (GPx), and peroxiredoxin (Prx); or non-enzymatic antioxidants including vitamins C and E, and GSH (36). In teleost fish, antioxidant enzymes are similar among different vertebrate species, where the main variability is in distribution of these enzymes in different tissues (36). Hydrogen peroxide (H_2_O_2_) is produced by leukocytes as part of their oxidative burst response involved in host defense (62, 91, 92). H_2_O_2_ can modulate gene expression through transcriptional factors and signaling molecules to regulate the late phase of leukocyte recruitment for inducing the production of the chemokine CXCl8 (93). In addition, H_2_O_2_ may regulate epigenetic modifications of transcriptional factor accessibility to CXCl8 and other genes encoding inflammatory factors.

Throughout evolution, higher oxygen capacity has become favored in fish based on their high activity levels. Fish cells may favor antioxidant protection in order to continue other important metabolic functions (36). Feeding and starvation conditions display the mediation of oxidative-antioxidant balance through the promotion or inhibition of ROS. Schvezov et al. (94) reported that rainbow trout (*Oncorhynchus mykiss*) after one feeding had molecular changes in oxidative defenses over a 96-hour post-feeding period (94). The highest antioxidant enzyme activities were found in the liver, followed by the intestine and stomach, with the lowest activities observed in the gills (94, 95). Interestingly, CAT was found in all tissues except for the liver, in contrast to GPx, which had the highest activity in the liver (94), suggesting that there may be interorgan relationships in antioxidant defense in the liver to favor oxidant-antioxidant balance. During an immune response, the liver has special mechanisms to scavenge ROS as well as synthesizing proteins like lipoproteins, such as very low-density lipoprotein (VLDL), low-density lipoprotein (LDL), and high-density lipoprotein (HDL), regulating intracellular protein turnover, producing bile acid, and synthesizing GSH (96–98). In order to perform these biomechanisms, the liver needs to take up the AAs, including: glycine, methionine, and proline (97). The bile can be excreted into the duodenum through the bile duct in response to a digestion-induced increase in the concentration of bicarbonate from ductal epithelial cells (97). Bile acids have the ability to conjugate with glycine and taurine to form bile salts to facilitate intestinal absorption of dietary lipids, including lipid-soluble vitamins like the antioxidant vitamin E to mediate antioxidant balance (26, 99). Lowered CAT activity in fish liver has been observed during starvation while SOD and GPx activities have been reported to increase, leading to oxidative damage (94, 100–102). A low SOD/GPx ratio will give better protection against oxidative stress because the efficiency of SOD is relatively higher for short periods of stress due to the balance between free radical production and antioxidant enzyme activity (94). To modulate oxidant-antioxidant balance with an immunostimulant supplement, one should be aware of the synergistic cooperation between immune response and the enzymes SOD, CAT, and GPx.

GPx is the main molecule in the response to reduce lipid hydroperoxides to their corresponding alcohols, thereby protecting the cell against lipid peroxidation (26). Stress has been shown to increase the activity of GPx in Atlantic salmon and rainbow trout 12 h post-exposure to 15 min of hypoxia stress from overcrowding (103). Interestingly, GPx activity may be affected by cortisol, a circulating glucocorticoid hormone that is a common biomarker for stress across various animal species. This result indicates that enzymes with antioxidant properties can be triggered in a stress response (103). Elevated concentrations of cortisol may induce the production of ROS, but cytokines produced from inflammation can act like regulatory molecules to facilitate immunosuppression (103, 104). Teleost fish are constantly exposed to various environmental stressors that can lead to oxidative stress, causing an imbalance of ROS, making it challenging to control the amount of free radicles through the ingestion of dietary antioxidants (103, 105).

#### 2.2.2 Oxidative stress and immune response

Exposure to stress, especially throughout multiple stages of life, can lead to chronic oxidative stress, which may induce morphological changes that can affect fish metabolism, including digesting and absorption (103, 106). It was observed in Atlantic salmon (*Salmo salar*) and rainbow trout (*Oncorhynchus mykiss*) that high stocking densities had a decrease in the integrity of the intestinal mucosal barrier and decreased mRNA expression of interferon gamma (IFNγ), the type II interferon and regulator of the gut immune response (103, 107). Chronic oxidative stress can cause damage to cell proteins, leading to cell death. Cells can modify the pH of their surrounding environments based on nutrient availability for host defense (108). Most eukaryotic cells have a pH ranging from 7.2 to 7.4 at a resting metabolic state, then decreasing during an immune response to denature pathogenic proteins (108, 109). In phagocytosis, lysosomes have a pH5, although it can vary depending on the cell type and environmental stressors (108, 110). Vacuolar-type ATPase (V-ATPase) enzymes have been identified as the key mediators of organelle acidification, which use ATP to drive protons into the lumen and the rate can impact the generation of ROS (108, 111, 112). To achieve antigen presentation and activate the adaptive immune response, the acidic phagolysosomal system promotes the net export of protons outside of the cell through the inner membrane antiporter, GadC (108, 113). This transporter relies on intracellular glutamate for the production of γ-aminobutyric acid (GABA; an inhibitory neurotransmitter) and, under acidic conditions (e.g., < pH 6.5), exchanges intracellular GABA for extracellular glutamate. Animals also require basic AAs (e.g., arginine, ornithine, histidine, and lysine) to maintain gut homeostasis through biochemical pathways to maintain low pH in the gut (108, 113, 114). These exemplify the relationship between acidic intracellular compartments for activating components of adaptive immune response by presenting antigens to T cells and inducing their activation for downstream defenses.

Oxidative stress can alter the composition of the gut microbiota, resulting in unwanted inflammation that can lead to oxidative damage from ROS. Environmental stress like water pollution from antimony have been investigated in zebrafish (66). Toxic metals, like antimony, in high concentration can alter the microbiota and affect both immune response and nutrition metabolism (64, 115, 116). The enzymes SOD, CAT, and GPx are critical to detoxify organs after toxic metal exposure because the metals produce ROS (64, 117). Acetylcholinesterase (AChE) is an enzyme used as a biomarker to quantify environmental pollution because it degrades acetylcholine, a neurotransmitter (64). Under stress, ROS in excess can inhibit AChE, meaning nerve signals are excited and oxidative stress will not be alleviated due to the accumulation of acetylcholine (64). This suggests that the inhibitory effect on AChE activity when ROS production is high can decrease the total antioxidant capacity (T-AOC) of the cell (64). T-AOC assays can directly reflect the capacity of nonenzymatic and enzymatic systems in response to environmental stressors. Malondialdehyde (MDA) is a biomarker for oxidative stress that can be used with T-AOC assays because it is a product of lipid peroxidation, typically for polyunsaturated fatty acids (64, 117, 118). MDA can induce damage to the structure of biofilms of pathogens and affect their normal function leading to proapoptosis (42, 119). In zebrafish, MDA content in the intestine increased with exposure time of antimony due to the oxidative damage from ROS (64). With excessive ROS production, SOD may be highly active convert superoxide anion to hydrogen peroxide, and CAT activity may be enhanced for H_2_O_2_ removal to resist oxidative damage (66, 120, 121). As exposure time progresses, the excess H_2_O_2_ will all be removed, and CAT activity will decreases to equilibrium (64). Thus, antimony stress results in dysbiosis of the zebrafish antioxidant system, ultimately impacting immune response and potential gut commensal bacteria.

Although quantifying the three main antioxidant enzymes, SOD, CAT, and GPx is beneficial to examine the effect of T-AOC, a histological marker of interest is the melanomacrophages centers (MMCs). MMCs are phagocytes containing lipofuscin, melanin, and haemosiderin found in the liver, head kidney, spleen (122, 123), and skin (124). These are believed to function in both immune defenses and can potentially orchestrate immune responses as well as oxidative stress. Researchers can determine AA metabolism, nucleotide synthesis, glycolysis, tricarboxylic acid (TCA) cycle, and oxidative phosphorylation (OXPHOS) while quantifying expression for fatty acid oxidation (125). An example is an OXPHOS pathway called glutaminolysis, which sustains T cell response through important enzymes GLS1 (encoding the K-type mitochondrial glutaminase) and GLUD (encoding the mitochondrial glutamate dehydrogenase); and if glutamine is deprived, phosphorylation is reduced in the T cells. This results in spleen lacking T cell proliferation (125). T cell immunity can determine the outcome of the adaptive immune response through the need for B cell activation and the direction of other effector mechanisms.

### 2.3 Adaptive immune response

In teleost adaptive immunity, lymphocytes are activated by innate immunity or the antigen itself binding to the lymphocyte antigen receptor, producing a specified response to eliminate pathogens and affording memory protection (13). This activation begins at peripheral lymphoid tissues, including GALT, where naïve lymphocytes first encounter antigen to perform either cell-mediated immunity or humoral immunity (114). Cell-mediated immunity starts with naïve T cells, lymphocytes that have not been exposed to antigen that is specific for their antigen receptor. Naïve T cells will circulate through the blood and peripheral lymphoid tissues in search of the antigen of the pathogen, which will be presented by proteins of the major histocompatibility complex (MHC) on APCs such as dendritic cells (29, 114, 126). This specific antigen and co-stimulation will then activate the naïve T cells and spark proliferation and differentiation into effector cells such as cytotoxic T cells (CD8+) or helper T cells (CD4+), which will clonally expand and secrete cytokines and chemokines (29). Cytotoxic T-cells can target and kill infected cells via antigen presentation from MHC class I on the cell surface, where MHC class I high allelic polymorphism provides broad pathogen recognition at the host population level (114, 127). T-helper cells have diverse functions based on the cytokines or chemokines produced (114). For example, T-helper 1 cells (Th1) activate infected macrophage microbicidal functions via phagocytosis by antigen presentation by MHC class II. Th1 cells during an inflammatory response can secrete proinflammatory cytokines including IL-1β, TNFα and IFN-γ, that will activate M1 macrophages that will kill the pathogen (128). In contrast, during an anti-inflammatory processes, Th2 cells activate M2 macrophages that secrete cytokines IL-4 and IL-10 and synthesize polyamines to promote wound healing and defense against helminths (128, 129). Another noteworthy Th cell are Th17 cells that stimulate neutrophils for inflammatory responses, as well as maintaining homeostasis at the epithelial barriers (29, 114).

Teleost fish have B lymphocytes that produce immunoglobulins of three isotypes: IgM, IgD, and IgT/IgZ. Antibodies are immunoglobulins that are synthesized and secreted by plasma cells (30, 130–132). IgM are the primary immunoglobulin present and can also be found in the serum, while IgT/Z are mucosal-specific immunoglobulin analogous to mammalian IgA and is primarily found in the intestine and skin mucosal tissue (13, 114). Nomenclature was standardized to IgT from IgZ recently (133) although some teleost’s clades such as catfish lack the isotype signifying other potential mucosal immune defense mechanisms (134).

Lymphocytes proliferate with intense protein synthesis and, therefore, a sufficient bioavailability of AAs is important metabolic processes (including ATP production, oxidation, antioxidant defense, and the production of antibodies and cytokines) and immune responses (26). Protein synthesis requires a large amount of energy, with the minimum energetic cost estimated to be 40 to 50 mmol ATP equivalents per gram of protein synthesized (26, 135–137). In growing fish, about 20 to 30% of dietary AAs are used for protein accretion (138, 139). By combining knowledge from nutrition and immunology, we can develop immunostimulants through AA supplementation to prevent the damage caused by ROS, while still promoting effective immune responses when fish are infected. There has been extensive research done on mechanisms that regulate the antioxidant defense system in mammals but not with teleost fish. Some of the reasons are experimental limitations from the amount of serum or plasma in juvenile fish, as well as challenges to isolate the leukocytes of fish to measure ROS and NO production. It is critical to find solutions to using minimal amounts of samples to take these quantitative measurements to evaluate the health and welfare of these fish within aquaculture.

## 3 Immunonutrition

### 3.1 Protein metabolism and cellular AA requirements

The nutritional requirements for AAs depend on numerous factors including the species, developmental stage, physiological status, gut microbiota, environment, and diseases (27, 140–143). Both immune response and antioxidant activity can be modulated through dietary supplementation as a non-invasive approach for controlling infectious diseases and minimize stress (21, 98, 144, 145). The teleost intestine has a plethora of digestive enzymes that facilitate the breakdown of complex nutrients into simple molecules that are absorbed into the intestinal mucosal cells by transporters. This process can regulate the downstream activation of the immune response through direct or indirect metabolic pathways. Understanding of their regulatory properties in the immune system in relation to AA requirements is imperative because protein feedstuffs contain different composition of AAs (138). For example, plant protein as the sole protein source in fish diets increases the risk of deficiency in important AAs like taurine, a sulfur-containing AA with antioxidant and osmoregulatory properties (138).

When using an AA as a supplement to aid immunity, it is important to understand its metabolic processes, including degradation in the gut, that will in turn affect the concentration of the AA in blood circulation, thus impacting dietary requirements (84, 98). To better understand this, we must evaluate digestibility and bioavailability of AAs prior to establishing an effective nutritional program for teleost fish. Digestibility of dietary protein is a percentage of AAs released from protein in the diet by the small intestine due to the actions of proteases and peptidases (84). This process is crucial because dietary proteins have no nutritional value unless hydrolyzed to free AAs and small peptides in the gut (84, 146, 147).

For fish, the dietary requirement for protein ranges from 30-60% of dietary dry matter, which is much greater than that for terrestrial mammals and birds (98). However the whole-body AA composition is largely similar among animals including fish, pigs, and chickens (138, 148, 149). For omnivorous teleost fish, their protein requirement is lower compared to carnivorous fish, and therefore the digestibility of plant-sourced feedstuffs is also important to consider because they generally have anti-nutritional factors including protease inhibitors (138, 150, 151). As mentioned previously, sustainable farming techniques are used in the aquaculture industry, including the use of plant-sourced feedstuff (such as soybean meal) instead of fish meal (84). In many regions of the world, the main plant-sourced ingredient is soybean meal; however, some teleost fish can have a limited capacity to use its protein because of a reduced activity of proteases in the digestive tract (53, 152). Important anti-nutrients present in soybean meal include not only protease inhibitors but also lectins, phytic acid, saponins, phytoestrogens, antivitamins, and allergens (150). There are two groups of protease inhibitors in soybean meal (trypsin inhibitors): Kunitz-type (153) and Bowman-Birk type 1 that reduces the digestibility of dietary protein (138, 154). These protease inhibitors can affect protein utilization by reducing trypsin activity in the small intestine by 30% (150, 155). To combat the effects of antinutritional factors, dietary supplementation with proteases can improve the digestibility of soybean meal in rainbow trout (138, 156).

The use of fishmeal has rapidly declined due to the serious environmental damages from high phosphorus content in fish fecal matter, creating poor water quality (157). Soybean meal is considered the most economic and environmentally friendly; however, compared to fishmeal, soybean meal is deficient in some AAs (e.g., glycine and methionine) and lacks taurine (158). Song et al. (157) performed a feeding trial in juvenile starry flounder (*Platichthys stellatus*) investigating differences in growth performance between diets with the protein component being fish meal or soybean hydrolysates (an improved soy protein with enzymatic modifications). They found that the soybean hydrolysates could replace part of fish meal in the diet without affecting the growth performance of the fish (157). This led to new initiatives of nutritionists within aquaculture to further understand protein metabolism in the context of fish health.

Intracellular protein undergo various cellular processes that regulates cellular integrity through protein synthesis and degradation (110). Proteases are separated by the membrane to control the rate of degradation during an immune response, like phagocytosis. Under stress, the lysosomal-vacuolar pathway is the dominant process for protein turnover in the liver, but other cells may rely on non-lysosomal proteolytic pathways (110, 159). Secretory factors from the duodenum, such as secretin produced by the S-cells, regulate secretions from the stomach, pancreas and liver, and cholecystokinin (CCK) by I-cells to stimulate the secretion of pancreatic digestive enzymes (84, 98, 147, 160). CCK aids in lipid digestion by first stimulating the gallbladder to contract and release the stored bile salts into the intestine, then signaling for enhancing the secretion of pancreatic digestive enzymes (84, 147). It is important to note that there are upper limits to dietary AA intake and that excessive AAs (e.g., > 2% glutamine and > 3% arginine in corn- and soybean meal-based diets for growing pigs) can become toxic (158). For example, supplementation with excessive methionine (e.g., 0.17% of diet) can reduce the life span in rats (158, 161). In addition, dietary supplementation with 563 ppm canavanine (a plant metabolite analogous to L-arginine) can reduce weight gain and feed intake in chicks (162). By contrast, dietary supplementation with 4% glutamate is safe for growing pigs (158).

### 3.2 AA Immunostimulants and intestinal immunity

Historically, AA requirements and functions in fish species have been determined primarily based on dose-response trials utilizing different protein sources. However, different feedstuffs have very different compositions of AAs (84, 147, 163, 164). Fatty acids and glucose can be stored in the body as triacylglycerols and glycogen, respectively, and when not utilized, they can accumulate and eventually impair the function of cells and tissues (138). Compared to mammals, fish liver and kidneys have high AA oxidation, but fish metabolism is more energetically efficient because they can excrete ammonia directly into their environment without needing ATP (26, 138, 141, 147). There are nine AAs that are not synthesized from any dietary AAs and have traditionally been regarded as nutritionally essential in all fish: histidine, isoleucine, leucine, lysine, methionine, phenylalanine, threonine, tryptophan, and valine (84). It remains unknown whether fish can synthesize arginine *de novo* (158). These AAs have a direct relationship with protein synthesis, so if one of the 9 nutritionally essential AAs is limited, it can impact intracellular protein synthesis and possibly proteolysis (26). Recently there have been suggestions to reevaluate the parameters for AAs because the term “non-essential” is a misnomer due to the critical role these AAs have in protein synthesis as well as immunity (84, 141, 147, 165). A widely accepted term, functional AAs, refer to AAs that not only participate in but also regulate key metabolic pathways. These AAs include arginine, glutamate, glutamine, glycine, and proline (138, 166, 167). Understanding optimal AA requirement in teleost fish cells is important for developing ideal diets and improving health through disease resistance, but only a handful of AAs have been extensively researched to investigates its health benefits. **Table 1** summarize the current AA supplementation studies performed in teleost fish. We will discuss arginine as well as glutamate and glutamine in this review for they may directly influence antioxidant balance.

**Table 1:** Effects of dietary supplementation with amino acids to improve health in teleost fish.

Amino Acid Supplement	Species	Health Benefits	Reference
Arginine	Channel catfish (*Ictalurus punctatus*)	Production of nitric oxide for the killing of pathogens	(168)
	Senegalese sole (*Solea senegalensis K.*)	Stress mitigation and innate immune response	(169)
	Rainbow trout (*Oncorhynchus mykiss*)	Hepatic metabolism to improve whole-body immune homeostasis	(170)
			(171)
L-Arginine	Nile tilapia (*Oreochromis niloticus*)	Lipid metabolism to improve whole-body immune homeostasis	(172)
Arginine and Citrulline	European seabass (*Dicentrarchus labrax)*	Inflammatory response	(173)
Arginine and Glycine	Rainbow trout	Growth performance and thyroid function	(174)
Arginine and Glutamine	Channel catfish	Disease resistance with vaccination	(20, 132)
Arginine and Glutamine	Hybrid striped bass (*Morone chrysops×Morone saxatilis*)	Growth performance and gut morphology	(175)
β-hydroxy-β-methylbutyrate (Leucine metabolite)	Hybrid striped bass	Growth performance and immune response	(176)
Carnosine	Rainbow trout	Antioxidant defense	(177)
Creatine	Hybrid striped bass	Muscle creatine levels	(178)
	Red drum (*Sciaenops ocellatus*)	Weight gain	(179)
Glutamate	Jian carp (*Cyprinus carpio J.)*	Lipid metabolism and antioxidant defense to improve whole-body immune homeostasis	(160)
Glutamate and Glutamine	Nile tilapia	Growth performance	(180)
Glutamine	Nile tilapia	Intestine morphology for mucosal immunity	(27)
	Gilthead sea bream (*Sparus aurata L.*)	Hepatic metabolism and antioxidant defense to improve whole-body immune homeostasis	(181)
	Jian carp	Growth performance	(182)
	Yellow catfish (*Pelteobagrus fulvidraco*)	Growth performance and disease resistance	(183)
	Channel catfish	Intestinal morphology to improve mucosal immunity	(132)
Glycine	Common Carp *(Cyprinus carpio)*	Growth performance	(184)
N-acetyl cysteine and glycine	Nile tilapia	Growth performance and antioxidant defense	(185)
Histidine	Red drum	Cataract-mitigating	(186)
Hydroxyproline	Salmon (*Salmo salar* L.)	Growth performance and bone composition	(187)
Isoleucine	Jian carp	Microbiota and antioxidant defense to improve mucosal immunity	(188)
	Hybrid grouper (*Epinephelus fuscoguttatus ♀ × Epinephelus lanceolatus ♂)*	Gut morphology for improving mucosal immunity	(189)
Lysine	Rainbow trout	Nitrogen and phosphorus excretion levels	(190)
	Turbot *(Scophthalmus maximus L.)*	Lipid metabolism to improve whole-body immune homeostasis	(191)
Methionine	Turbot	Growth performance	(192)
	Gilthead sea bream	Antioxidant defense to improve whole-body immune homeostasis	(193)
Methionine & Taurine	Largemouth bass *(Micropterus salmoides)*	Growth performance and feed efficiency	(138)
Taurine	Senegalese sole	Lipid metabolism to improve whole-body immune homeostasis	(194)
	Red sea bream (*Pagrus major)*	Hepatic metabolism to improve whole-body immune homeostasis	(195)
	Japanese flounder (*Paralichthys olivaceus)*	Growth performance	(196)
	yellowtail (*Seriola quinqueradiata)*	Growth performance	(197)
Taurine	Asian seabass *(Lates calcarifer)*	Antioxidant defense to improve whole-body immune homeostasis	(99)
	Spotted Knifejaw (*Oplegnathus punctatus)*	Growth performance and feed efficiency	(53)
Tryptophan	Rohu *(Labeo rohita)*	Stress mitigating to improve whole-body immune homeostasis	(198)
L-Tryptophan	Atlantic cod *(Gadus morhua)*	Aggression levels and stress mitigating to improve whole-body immune homeostasis	(199)
	Rainbow trout	Plasma cortisol levels and Stress mitigating to improve whole-body immune homeostasis	(200)

#### 3.2.1 Glutamine and glutamate

Glutamine is endogenously synthesized from glutamate and ammonia by glutamine synthetase, then deaminated into glutamate and ammonia by phosphate-activated glutaminase, meaning that both glutamine and glutamate play a valuable role in ammonia detoxification (138, 201, 202). Both glutamine and glutamate are required for ammonia detoxification in fish through the enzymes glutamine synthetase and glutaminase (GLS), demonstrating that there is a glutamine–glutamate cycle to regulate the intracellular concentrations of glutamine (138, 202). Both of these AAs are major sources of energy in the intestinal mucosa and glutamine can activate the mTOR pathway to initiate polypeptide synthesis (84, 147). Carvalho et al. (27) showed that 1.33% (dry matter basis) is the most optimal amount of glutamine in the diet for the immune function of juvenile Nile tilapia (27). Through histological analysis, the proximal intestine, villus height was higher and the ratio of villus:crypt was higher (27). GSH is an antioxidant molecule that participates in the teleost intestinal mucosal immune response by protecting the epithelium from oxidative damage caused by pathogens. GSH also improves the functionality of enterocytes to repair, proliferate, and recruit immune cells (84). Carvalho et al. (27) found that dietary supplementation with up to 1.71% of glutamine increases GSH concentration in the intestine, which is essential to prevent oxidative stress and adverse health consequences (27). This suggests that oxidative damage can be associated with inflammatory molecules, including changes in the expression and/or activities of antioxidative enzymes in reflection of oxidative stress. Glutamine supplementation may also be required to support immune responses in tilapia by serving as a precursor for glutamate, thereby mediating GSH synthesis and scavenging ROS to maintain cellular redox balance (27, 185).

When T cells are activated to combat an infection, the transporters ASCT2 and SNAT2 are upregulated to increase glutamine availability for the formation of glutamate in the mitochondria by GLS (125, 203). It will then be converted to alpha-ketoglutarate (AKG) by glutamate dehydrogenase (GLUD) through a metabolic process called glutaminolysis to promote cell proliferation, protein synthesis, and maintain redox balance (125, 204, 205). AKG is a precursor to glutamine and an intermediate of the TCA cycle that may play a role in antioxidant capacity during an immune response (42). The TCA cycle can facilitate NADPH production and provide extra ATP to the intestinal mucosal cells by promoting electron transport in the respiratory chain and maintaining the activity of the ion transporters (125, 206–208). This reduces the electron leakage from intracellular sodium/calcium ion loading and slows the formation of free radical damage (125, 209). Exogenous AKG promotes the absorption of nutrients through modulating intestinal AA metabolism by activating NF-κB to favor upregulation of expression for pro-inflammatory cytokines (46, 125, 210–213). NF-κB signaling in epithelial cells maintains immune homeostasis in mucosal tissues, and when in a resting metabolic state, NF-κB is complexed with an inactive inhibitory protein IκB-α (125). During oxidative stress, inflammatory signals can activate the enzyme IκB kinase (IKK) to phosphorylate the IκBα protein, which causes ubiquitination (125, 203). Ubiquitination is an ATP-dependent process that is highly regulated for homeostasis, and the dissociation of IκB-α from NF-κB can cause degradation of IκB-α which will activate NF-κB to be translocated into the nucleus (125, 214). It has been reported that commensal bacteria may have the ability to activate NF-κB in epithelial cells by stimulating pattern recognition receptors including TLR and NLRs (125, 215). For example, TLR4 recognizes inflammation to then activate the myeloid differentiation factor 88 (MyD88)-dependent pathway in Th cells that will release inflammatory cytokines (42, 216, 217).

Wu et al. (42) investigated effects of dietary supplementation with 1% AKG in Songpu mirror carp as an alternative to AAs to modulate immune response. They challenged the mirror carp with *Aeromonas hydrophila* and witnessed abnormal swimming behavior where the carp sought more oxygen on the water surface (42). Increasing oxygen needs was consistent with the data mRNA expression indicating a systemic immune response due to stress. Within intestinal tissues, they observed TLR4b intracellular signal transduction through a MyD88-independent pathway that upregulated expression of inflammatory cytokines TNF-α, IL-1β, IL-6 and IL-8 in addition to down regulating claudins and ZO-1 (42). Claudin-1, claudin-3, claudin-7, claudin-11 and zonula occludens-1 (ZO-1) were upregulated exhibiting that AKG supplementation could increase activities of tight junction structural protein genes in intestinal mucosal cells to promote integrity and reduce intestinal permeability. Oxidative stress in the intestine was quantified through the enzyme CAT while total antioxidant capacity used the MDA biomarker, and both demonstrated a decrease in content during AKG supplementation (42). AKG could reduce oxidative damage caused by *A. hydrophila* infection by increasing antioxidant capacity promoting scavenging radical properties. By inhibiting the generation of oxygen free radicals, it prevents lipid peroxidation as well as tissue damage (42, 218). AKG is capable of promoting expression of claudins under stress and can alleviate the stress response by reshaping the cytoskeletal microfilaments through the myosin light chain kinase (MLCK) signaling pathway (42, 218–220). MLCK functions in tight junction permeability by regulating the protein abundances of claudins, occludins, and zonula occludens (ZOs) (42). AKG supplementation in Songpu mirror carp can improve the contraction of the actin ring before the tight junctions, stabilizing the tight junction proteins and their surroundings cytoskeletal protein distributions to reduce intestinal permeability (41, 42, 221, 222). Protection of tight junction structure of intestinal epithelial cells during an infection increases intestinal mucosal permeability, therefore activating intestinal mucosal immune response. There are other AAs and associated metabolites that can enter immune cells through various junctions (158). However, these junctions and transporters need to be extensively studied for each AA.

#### 3.2.2 Arginine

Another AA of interest for modulating immune responses is arginine, a nutritionally essential AA required by teleost fish for growth and immunity (26, 223). Arginine-derived NO is a cytotoxic molecule of macrophages that mediates inflammation (84, 147), where inflammation can be categorized as acute or chronic. Arginine is abundant in tissues and through the production of NO by macrophages, it can facilitate an immune response (26, 84, 147). NO is a cytotoxic molecule of macrophages that mediates inflammation through the production of cytokines that can directly or indirectly affect gene expression (84, 141, 147, 167, 224). In teleost fish, there is likely limited endogenous arginine synthesis due to the low activity of the enzyme carbamoyl phosphate synthase III and pyrroline-5-carboxylate synthase (84, 147, 225, 226). In contrast to most mammals, teleost fish may not synthesize citrulline from glutamate, glutamine, and proline but can produce citrulline from arginine via NO synthase at a very low rate (26, 84, 147). Hoseini et al. (227) investigated the effects of dietary arginine supplementation on systemic immune responses in common carp that were challenged with ammonia stress (227). They observed that arginine supplementation reduced ammonia levels in the plasma, elevated plasma glutamate and declined glutamine is due to lower available ammonia in the arginine-treated fish (227). After ammonia exposure, plasma arginine decreased in fish fed both the control and the arginine supplemented diets, but paradoxically the decrease in plasma arginine was greater in the arginine-supplemented group, possibly due to enhanced protein synthesis in the whole body. Plasma glutamate and glutamine decreased after exposure to ammonia, which could be due to the reaction of glutamate with ammonia to form glutamine and the excretion of glutamine from the body to help the fish cope with ammonia toxicity (227, 228).

From these observations, Hoseini et al. (227) concluded that arginine supplementation may be beneficial for ammonia tolerance in common carp to facilitate detoxification through AA metabolisms (227). Hoseini et al. (229) also investigated effects of dietary arginine supplementation on the common carp that were exposed to density stress. They observed that both arginine-deficient or excessive diets had detrimental effects on fish health, causing cortisol elevation, innate immunity weakness, and down-regulation of cytokine gene expression. In addition, they observed that optimum arginine levels inhibited stress-induced cortisol secretion and lysozyme activity through the downregulation of expression of IL-1β and IL-8 genes. The interaction of dietary arginine levels and stocking density on plasma cortisol suggest that different stress types might affect arginine requirement in common carp potentially through different metabolic pathways (229). Optimum dietary arginine levels boosted fish health and suppressed stress signals by stimulating the expression of inducible NO synthase (iNOS) to produce NO (229, 230). iNOS also regulates the secretion of inflammatory cytokines through activation of guanylate cyclase, as well as the release of inflammatory cytokines by binding all subtypes of glutamate receptor (229, 231, 232). At low cortisol levels in the plasma, there may be higher cytokine gene expression for regulatory cytokines and polyamine production for arginine-dependent cell proliferation (229). This is consistent with the report that arginine may increase the resistance of channel catfish to *Edwardsiela ictaluri* by producing NO (138, 167, 233).

M1 macrophages are believed to metabolize arginine into NO through the action of iNOS resulting in a macrophage population with increased microbicidal activity (57, 128). M2 macrophages metabolize arginine by converting it to ornithine and further converting ornithine into polyamines through ornithine decarboxylase (ODC) and S-adenosylmethionine decarboxylase (SAMdc) (128, 234, 235). Between the two subtypes of macrophages, competition for arginine is a balance between inflammatory response and cellular repair, but the competition between the two macrophage subtypes can deplete the arginine pool, therefore making the organism more susceptible to disease (128, 236, 237). M1 macrophages depend on extracellular arginine levels and when there is a sufficient supply the cells exports citrulline; in contrast under depleted supplies the cells import citrulline (128, 238). This is done through the formation of polyamines, thereby regulating the inflammatory response through inhibiting inflammatory mediators, antioxidant properties, as well as cell proliferation (128, 239, 240). M2 macrophages directly convert arginine to ornithine for polyamine synthesis for tissue repair as a result of increases in the enzymatic activities of ODC1, ODC2, SAMdc1, and SAMdc2 in the liver and head kidney (128). In addition, polyamines are produced by exfoliated enterocytes to also scavenge ROS and protect cells from oxidative stress (240, 241). Intracellular polyamine oxidation leads to an increase in ROS that causes DNA damage in activated immune cells (240, 242, 243). More studies are needed to further understand how polyamines affect both protein synthesis and gene expression for immune proteins. Li et al. (223) reported effects of dietary 1% and 2% L-arginine supplementation for 8 weeks on intestinal homeostasis in tilapia. They observed altered composition, diversity, and functional profiles of gut microbiota with increasing α-diversity and altering β-diversity Li et al. (223). Through evaluating the enhanced mRNA expression for Pparα, acox1, and Pgc-1α in tilapia intestinal tissues, Li et al. (223) found that arginine may regulate lipid metabolism in the intestine (223). Li et al. (223) verified the activity by culturing IPEC-1 cells (small- intestinal epithelial cells) to determine lipid metabolism through starving the cells and using oleic acid to induce triglyceride accumulation (223). These authors found that L-arginine mediated the AMP-activated protein kinase (AMPK) signaling pathway and mRNA levels were measured for Acox 1 and Acox 2 (223). When extracellular arginine availability was limited, triglycerides accumulated in the cells. Through examining the sequence of the 16S ribosomal RNA gene, Li et al. (223) and Ren et al. (244) found that dietary L-arginine supplementation affected the composition of the intestinal microbiota and therefore the intestinal immune response (223, 244). Since the intestine catabolizes about 40% of dietary arginine, its supplementation inhibited lipid accumulation in the intestine as confirmed through BODIPY staining to reveal reduced triglycerides content (223, 245, 246). Elevated mRNA levels for Pparα, Acox1, and Pgc-1α were associated with increased fatty acid oxidation through the activation of the in the enterocytes (223). Thus, there may be a trade-off between growth and the immune response in which growth is hindered until the infection is resolved (128). Dietary arginine supplementation could improve the physiological processes of digestion and the absorption of nutrients from the diet, thus enhancing growth performance and immune responses (particularly intestinal mucosal immunity).

## 4 Discussion

Phagocytosis involves pathogen enclosure in a phagosome and its subsequent destruction inside this structure via the fusion with the lysosome or the action of ROS. Current knowledge of teleost mucosal immunity is based on studies performed on fish species in addition to other species belonging to different genera, families, and orders. The inherent differences make it challenging to consolidate these findings to identify a comprehensive landscape on gut mucosal response facilitated through AA supplementation by modulating oxidant-antioxidant balance. Systematic studies are necessary for a comprehensive understanding of immunity in all fish, but a lack of specific antibodies against these cell markers is a limiting factor for the study of immune cell subsets in most teleost’s (20). Whole genome sequencing information from phylogenetically diverse organisms can offer other tools by comparing immune receptors (43). Proteomic analysis can validate individual proteins in the intestine compared to systemic efficiency from phagocytosis (247). Primary cells lines also provide a valuable model, but to our knowledge no one has created a gut mucosa primary cell line. We hope that researchers will be successful in developing a gut mucosa primary cell line as well as mono- and poly-clonal antibodies for some teleost fish species to phenotype leukocytes and quantify cellular regulation of certain immune cell populations. An another powerful tool that can be used to analyze distinct gene expression datasets from the same experiment in samples of interest is called non-metric multidimensional scaling (NMDS) (128). This method has not been utilized to a large extent, but should be incorporated in future studies because the plots can display a clear separation between uninfected and infected groups, as well as enzymatic activities (128). Another appealing perspective would be to look further into the effects of both pathogen and AA supplementation on epigenetics, which is defined as the heritable alterations of gene expression through covalent modifications of DNA in addition to core histones without alterations in the DNA sequence (248). This process is initiated by S-adenosylmethionine-dependent DNA and histone methylation, histone acetylation by acetyl-CoA, histone phosphorylation, and histone ubiquitination (249).

In recent years, there has been growing interest in the role of dietary AAs in the immune responses of fish, especially their mucosal immunity. Adding crystalline AAs in diets for fish can effectively improve intestinal mucosal integrity, enhance growth performance, modulate intestinal microbiota, and improve resistance to infections (250, 251). To further understand teleost, gut mucosal immunity in response to dietary AA supplementation, future studies should involve a collaboration of genomics, proteomics, nutrition, immunology, macrobiotic and metabonomic analyses. The collaboration of different specialties in a single feeding trial, even for one teleost species can provide a more holistic understanding of the mucosal immunity in fish.

Amino acid supplementation has a promising future for its ability to modulate immune response through metabolic processes, oxidant-antioxidant balance, and physiological requirements (158, 164). While the immunonutrition field has made advances, only a few research teams have investigated cell- and tissue-specific energy requirements in gut mucosal immune responses. By bridging the knowledge of protein metabolism during oxidative burst or oxidative stress, nutritionists can develop sustainable aquafeeds through modifying AA composition to promote specific immune response, including gut mucosal immunity. More nutritional experiments are warranted to investigate the dosage effect and chronic impact of using AA as a dietary immunostimulant in fish immunity. An exploration of the response of fish at different stages to dietary AA supplementation in terms of their immune system development will also lay a solid physiological basis for the broad application of using AA to modulate fish immune systems. Rapid advances in the field of teleost gut mucosal immunity require more sophisticated tools, especially when utilizing the zebrafish model in the biomedical science field to understand biochemical mechanisms to find cure for inflammatory diseases. Overall, we propose further investigation on dietary supplementation with functional AAs to improve immune response. Availability of well-developed tools in aquaculture models will have major impacts on the provision of high-quality fish protein for global food security by understanding the molecular impacts on immune components in the gut as well as the cellular mechanisms for oxidant-antioxidant balance. Through research, we can compare various AAs and characterize their antioxidant benefits to make the aquaculture more sustainable by improving plant-based diets with amino acid supplementation to help fish within aquaculture resist pathogenic microbes to improve the growth and health of the aquatic animals.

## Author contributions

KH drafted the work for this review, contributing to majority of the interpretation of data for the work. MC provided approval for publication of the immunology content, while contributing and revising it critically for important intellectual content. GW also provided approval for publication of both the immunology and nutrition content. Both WH and GW also contributed to the nutrition and amino acid concepts, and all authors agreed to be accountable for all aspects of the work in ensuring that questions related to the accuracy or integrity of any part of the work are appropriately investigated and resolved. All authors contributed to the article and approved the submitted version.
